# Winter Hive Debris Analysis Is Significant for Assessing the Health Status of Honeybee Colonies (*Apis mellifera*)

**DOI:** 10.3390/insects15050350

**Published:** 2024-05-13

**Authors:** Ivana Tlak Gajger, Klara Bakarić, Ivan Toplak, Laura Šimenc, Urška Zajc, Metka Pislak Ocepek

**Affiliations:** 1 Faculty of Veterinary Medicine, University of Zagreb, Heinzelova 55, 10000 Zagreb, Croatia; 2 Institute of Oceanography and Fisheries, Šetalište Ivana Meštrovića 63, 21000 Split, Croatia; klara.zubak.novak@gmail.com; 3 Veterinary Faculty, University of Ljubljana, Gerbičeva 60, 1000 Ljubljana, Slovenia; ivan.toplak@vf.uni-lj.si (I.T.); laura.simenc@vf.uni-lj.si (L.Š.); urska.zajc@vf.uni-lj.si (U.Z.); metka.pislakocepek@vf.uni-lj.si (M.P.O.)

**Keywords:** honeybee colony (*Apis mellifera*), samples of winter hive debris, PCR/qPCR, *Paenibacillus larvae*, *Melissococcus plutonius*, *Crithidia mellificae*, *Lotmaria passim*, *Vairimorpha* spp. (*Nosema* spp.), Acute Bee Paralysis Virus, Black Queen Cell Virus, Deformed Wing Virus and Sacbrood Virus, *Aethina tumida*, *Varroa destructor*

## Abstract

**Simple Summary:**

Diseases are a major cause of honeybee colony weakness and death. An effective and fast way to diagnose subclinical infections is by sampling and analyzing debris from hive bottom boards. Molecular tests, like PCR and qPCR can be used to identify disease-causing agents quickly. In this study, we analyzed hive debris samples from Croatian apiaries to check the presence of pathogens, such as *Paenibacillus larvae*, *Melissococcus plutonius*, *Crithidia mellificae*, *Lotmaria passim*, *Vairimorpha* spp. (*Nosema* spp.), *Aethina tumida*, Acute Bee Paralysis Virus (ABPV), Black Queen Cell Virus (BQCV), Deformed Wing Virus (DWV) and Sacbrood Virus (SBV). Debris samples were also examined to quantify *Varroa destructor* mites, and natural mite fall was observed in spring. Many honeybee colonies were infected with four to six agents, which is probably why some colonies failed to survive winter.

**Abstract:**

Honeybee diseases are one of the most significant and most common causes of honeybee colonies’ weakness and death. An early diagnosis of subclinical infections is necessary to implement precautionary and control measures. Sampling debris from hive bottom boards is simple, non-invasive, and cheap. In this study, we collected winter debris samples in apiaries located in the continental part of Croatia. We used molecular methods, PCR and qPCR, for the first time to analyze those samples. Laboratory results were compared with the health condition and strength of honeybee colonies at an apiary in spring. Our study successfully identified the presence and quantity of various pathogens, including the presence of *Vairimorpha* spp. (*Nosema* spp.), quintefied *Paenibacillus larvae*, Acute Bee Paralysis Virus (ABPV), Black Queen Cell Virus (BQCV), Deformed Wing Virus (DWV), and Sacbrood Virus (SBV). However, our analysis did not detect *Melissococcus plutonius*, *Crithidia mellificae*, *Lotmaria passim*, and *Aethina tumida*. Samples of winter debris were also examined for the presence and quantification of the *V. destructor* mites, and their natural mite fall was observed in spring. Honeybee colonies were simultaneously infected by an average of four to six pathogens. Some observed honeybee colonies developed characteristic symptoms, while others did not survive the winter.

## 1. Introduction

Honeybees, solitary bees, bumblebees and many other pollinating insects not only act as biosensors for the health of natural ecosystems, and play a key role in maintaining biodiversity, but they also provide food for human and animal consumption [1]. Although several species of pollinator insects are necessary for the pollination of plants, the most economically important is the European honeybee (*Apis mellifera* L.). It can make up to 80% of the entire pollinator population [2,3,4,5], depending on the number of honeybee colonies and wild pollinators in some geographical areas. It is known that the number of wild pollinators is declining worldwide, making pollination by honeybees increasingly important to produce quality fruits with a nicer appearance, greater mass, and longer shelf life, resulting in a higher market value [6]. Accordingly, it is estimated that the economic value of honeybee pollination is several dozen times higher than the value of apian products. Since one-third of the human diet depends directly or indirectly on pollination, the health of honeybees and other pollinating insects is crucial for the global food supply [7].

Troubling facts, like inexplicably high losses of honeybee colonies in certain countries and a significant increase in reports of honeybee colony mortality across the USA and EU since winter 2006–2007 [7], when beekeepers recorded losses of up to 30% [8], have triggered and increased the interest of the scientific community and the public. However, many years of intensive research and loss monitoring [9,10,11,12,13,14] have not revealed a specific cause of honeybee colony decay, which is why it is thought to be caused by the simultaneous action of several pathogens and exposure to other adverse environmental factors. Influences such as habitat loss, pesticide use, pathogens, climate change, and local weather conditions act simultaneously and cause increased susceptibility of honeybee colonies to disease [1,15,16,17]. Although the cause is different, depending on the region or geographical area, dead honeybee colonies are most often associated with a high degree of infestation with parasitic mite *Varroa destructor* and *Vairimorpha* spp. (*Nosema* spp.), in combination with other pathogenic microorganisms [18,19,20,21,22,23,24,25]. The spread of honeybee diseases due to robbing or the drifting of adult bees increases depending on the density of colonies in the geographical area. When determining the occurrence of an economically significant disease, it is important to prevent the spread of infection and limit transmission to new areas, by early detection of the disease’s causative agents [26]. 

Collecting hive debris is non-invasive, easy to perform and is accessible also in winter months. This allows detection of subclinical levels of pathogens and analysis and assessment of the health status of honeybee colonies before spring and the appearance of clinically visible signs. Consequently, it accelerates early detection and timely eradication or control of diseases. Moreover, in some cases, by applying simple technological measures we can prevent the appearance of clinical signs/forms of the disease and thereby reduce harmful economic losses for beekeepers. For this reason, early diagnosis of subclinical levels of pathogenic microorganisms in the hive or apiary is a priority for effective disease prevention in beekeeping practices [27]. 

Although there is still insufficient knowledge about the efficiency and consistency of the determination and quantification of many pathogenic causative agents from winter hive debris samples [27], there are some areas/pathogens (e.g., *Paenibacillus larvae*) that are well studied [28,29]. In this regard, the goal of this work was the collection of samples of winter hive debris from six different apiaries located in the continental part of the Republic of Croatia, determining by PCR/qPCR the presence of economically important pathogens (*P. larvae*, *Melissococcus plutonius*, *Crithidia mellificae*, *Lotmaria passim, Vairimorpha* spp. (*Nosema* spp.), Acute Bee Paralysis Virus (ABPV), Black Queen Cell Virus (BQCV), Deformed Wing Virus (DWV) and Sacbrood Virus (SBV), *Aethina tumida*, *V. destructor*), and estimation of how well the results relate to the actual health status of honeybee colonies in spring, when clinical examinations were carried out.

## 2. Materials and Methods

### 2.1. Sampling Locations

In February 2022, hive debris samples were collected on six randomly selected apiaries located in the continental part of the Republic of Croatia. Two apiaries were in the Sub-Sljeme zone of the County of Zagreb: Apiary 1 (A1) in Gračani (GPS coordinates: N 45°86’32″; E 15°98’17″ with 40 hives of type Alberti-Žnideršič (AŽ), and Apiary 2 (A2) in the village of Markuševac (GPS coordinates: N 45°87’39″; E 16°01’82″) composed of 20 beehives in the Langstroth Root (LR) type. Three apiaries belong to Koprivnica-Križevci County: Apiary 3 (A3) was located in Poljana Križevačka (GPS coordinates: N 45°58’11″; E 16°32’09″) and consists of 306 hives of type LR, Apiary 4 (A4) was located in the municipality of Sveti Ivan Žabno (GPS coordinates: N 45°95’19″; E 16°62’48″) consisting of 50 hives of type LR, and Apiary 5 (A5) in the municipality of Sveti Petar Orehovec (GPS coordinates: N 45°87’39″; E 16°01’82″) with 56 hives of type LR. Apiary 6 (A6) was located in Varaždin County (GPS coordinates: N 46°28’33″; E 16°31’77″), with 110 LR-type hives.

In each apiary, honeybee colonies were randomly selected for sampling. From A1, we collected ten samples, from A2, A3, A4 and A6 five samples each, and from A5 nine samples. A total of 39 samples were taken with plastic spatulas, stored in plastic bags and marked with the number of the hive and name of the apiary, and then delivered to the laboratory. Each hive debris was divided into three parts and stored accordingly until use, a part for DNA extraction stored at a temperature below −18 °C, a part for RNA extraction at −70 °C, and the third part for the determination of *V. destructor* mite numbers at 4 °C. Due to winter conditions at the time of sampling, clinical inspections of honeybee colonies were carried out in spring.

### 2.2. DNA Extraction

From each hive debris sample, 0.5 to 3 mL of material was taken, transferred into a disposable Eppendorf tube, and supplemented with distilled water at a ratio of 1:9 (4.5 mL to 27 mL). The samples were then processed according to a previously described protocol [28] and, from each sample, 1 mL suspension was centrifuged at 10,000× *g* for 5 min and the obtained pellet was stored at −33 °C until DNA extraction.

For the determination of *C. mellificae*, *L. passim*, *A. tumida*, *M. plutonius*, *P. larvae* and *Vairiomorpha* spp. (*Nosema* spp.), DNA was extracted using iHelix Complex kit (Institute of Metagenomics and Microbial Technologies, Ljubljana, Slovenia; https://www.ihelix.eu/ (accessed on 20 of March 2024) according to the manufacturer’s instructions. In detail, a 392 μL lysing buffer (D-buffer) was added to each sample along with 8 μL of proteinase K (20 mg/mL). After shaking the samples at 6400 rpm for 45 s, they were incubated for 15 min at 56 °C (2×) and 10 min at 100 °C (1×). Then, each sample was centrifuged at 10,000× *g* for 5 min and supernatant was obtained from which approximately 350 μL was transferred to new sterile 2 mL micro-centrifugal tubes. A triple volume of a binding buffer (B-buffer) was added to allow optimal DNA binding to the silica membrane of the spin-column system. For nucleic acid purification, samples were washed with 600 μL and then with a 500 μL rinse buffer containing ethanol (W-buffer). The addition of a 100 μL eluting buffer isolated purified DNA stored at −18 °C until the next phase of the examination.

### 2.3. Testing of Samples Using Molecular Method PCR/qPCR

#### 2.3.1. Identification of *Vairimorpha* (*Nosema*) Species Using PCR Test

The PCR method with specific oligonucleotide primers (Table S1) was performed according to Fries et al. (2013) [30]. Visualization of amplified PCR products was performed using the QIAxcel electrophoresis system with a QIAxcel DNA high-resolution kit (Qiagen, Hilden, Germany). The analysis was processed using the QIAxcel Screen Gel software system 1.5.0. The size of the segments of each product was determined by comparison with the size marker (50 bp–800 bp, Qiagen, Hilden, Germany) and alignment marker (15 bp–1 kbp, Qiagen).

#### 2.3.2. Triple TaqMan Real-Time PCR for Determination of *Crithidia Mellificae* and *Lotmaria Passim*

Isolated DNA was multiplied using a real-time PCR method and specific oligonucleotide primers (Table S2) according to Xu et al. (2018) [25]. The 18S rRNA Hymenoptera gene was incorporated into the reaction as an internal control that allows accurate detection of PCR inhibition and DNA extraction failure for the correct interpretation of negative results. The cytochrome b gene was applied to distinguish between *C. mellificae* and *L. passim*. In detail, the TaqMan real-time PCR test was performed in a triple format with reaction volumes of 25 μL. The reaction mixture consisted of 2.5 μL isolated DNA, 12.5 μL master mix (Maxima Probe qPCR Master Mix (2X), with separate ROX vial, Thermo Scientific™, Waltham, MA, USA), 0.1 μM of each primer for Hymenoptera 18S and *L. passim*, 0.2 μM of each primer for *C. mellificae*, 0.3 μM of each probe for *L. passim* and *C. mellificae*, 0.2 μM probes for Hymenoptera 18S, 0.03 μM ROX, and ultra-pure water. Positive controls were prepared using internal positive samples, while negative controls were made with ultra-pure water. The primers and TaqMan probes used in the analysis are shown in Table S3. The PCR was performed on the AB7500Fast device (Applied Biosystems by Thermo Fisher Scientific, Foster City, CA, USA), and the program had the following stages: initial at 95 °C for 10 min, followed by 40 two-step amplification cycles, each consisting of 15 s denaturation at 95 °C and one min binding at 60 °C.

#### 2.3.3. Double Real-Time PCR to Determine *Melissococcus plutonius* and *Paenibacillus larvae*

The analysis was conducted following a published protocol [31]. A positive sample was used as an internal positive control, and ultra-pure water was used as the negative control. The primer and probe information are available in Tables S4 and S5. Amplification was performed on an AB7500Fast temperature circle device (Applied Biosystems by Thermo Fisher Scientific, Foster City, CA, USA) using the program, which began with 10 min at 95 °C, followed by 40 two-step amplification cycles of 15 s at 95 °C and 60 s at 60 °C.

#### 2.3.4. TaqMan Real-Time PCR for Determination of *Aethina tumida*

The reaction mixture for the TaqMan PCR test consisted of 2.5 μL of isolated DNA, in which 12.5 μL of MasterMix was added containing 900 nM of each primer and 200 nM TaqMan probes, 0.03 μM ROX and ultra-pure water [32]. A known positive sample was used as a positive control and water as a negative control. The primers and TaqMan probes used in the test are shown in Tables S6 and S7. The PCR was performed on the AB7500Fast temperature circle device (Applied Biosystems by Thermo Fisher Scientific, Foster City, CA, USA) at 95 °C for 10 min, followed by 40 two-step amplification cycles, each consisting of 1 min denaturation at 60 °C and binding at 60 °C for 15 s [33].

### 2.4. RNA Extraction

Three debris samples from each apiary (in total 18 samples) were examined for the presence of nucleic acids of four viruses: ABPV, SBV, BQCV and DWV. One gram of each sample was transferred to Ultra-Turrax DT-20 tubes (IKA, Königswinter, Germany) with 5 mL RPMI 1640 media (Gibco, Paisley, UK) and incubated at room temperature for 30 min. After homogenization in the Stomacher device (BagMixer; Interscience, Paris, France) and centrifugation at 2500× *g* for 15 min, 2 mL of supernatant from each sample was stored at −70 °C until the next examination phase.

The total RNA was extracted from the 140 μL obtained suspension of each sample using the QIAamp Viral RNA Mini kit (Qiagen, Hilden, Germany). According to the manufacturer’s instructions, a 560 μL AVL buffer was placed into the 2 mL tubes, to which the RNA carrier was added. For precipitation of nucleic acids, 560 μL of ethanol was added and 630 μL of total suspension was transferred to QIAamp Mini columns with filter and centrifuged at 8000 revolutions over 1 min. Mini columns were placed in new test tubes into which 630 μL of the mixture was transferred and re-centrifuged at 8000 revolutions for 1 min. The samples were then washed twice using 500 μL flush buffers (AW1 and AW2 Buffer). Finally, 60 μL eluting buffer (AVE Buffer) was added, thus completing the isolation of the purified viral RNA of each sample, which was stored at −70 °C until further use.

### 2.5. Testing for Presence and Quantification of Viruses Using the RT-qPCR Molecular Method

The specific parts of genomes for these viruses were multiplied using the previously described quantitative real-time RT–PCR method (RT-qPCR) [34]. Reverse transcription was made in one step using the QuantiNova Pathogen +IC Kit (Qiagen, Hilden, Germany). The RT–PCR mixture consisted of 5 μL of QuantiNova Master Mix, 2 μL 10 × internal control (IC-Internal Control Probe Assay), 1 μL IC (1: 100), 4.5 μL deionized water, 1 μL upstream primer (200 nM), 1 μL downstream primer (200 nM) and 0.5 μL TaqMan probe (100 nM), and 5 μL extracted RNA of 20 μL of final volume. RT-qPCR was performed on the Mx3005P temperature circulation) (Stratagene, La Jolla, CA, USA), and the program included: the initial phase of reverse transcriptase at a temperature of 50 °C for 20 min and then the phase of denaturation and activation of the polymerase enzyme at a temperature of 95 °C for 2 min. This was followed by 45 repetitive cycles, each cycle consisting of a denaturation phase at 95 °C for 15 s, then a binding phase at 60 °C for 30 s and an elongation phase at a temperature of 60 °C for 30 s. For each virus, a specific pair of primers was used (Table S8).

For each RT-qPCR treatment for each virus, positive controls were included, which are prepared as suspensions of verified known samples of four different viruses. The negative controls consisted only of RPMI 1640 media. Standards with a known number of copies of viral RNA were prepared in a ten-fold dilution of 10^3^ to 10^7^ copies and added to each RT-qPCR processing (Tables S9 and S10). The exact number of viral RNA molecules in individual samples was determined from the standard curve for each of the four viruses individually. The results for each sample were analyzed using The MxPro-Mx3005P v4.10 software (Stratagene, La Jolla, CA, USA) and the exact number of copies was determined from the standard curve. The results were expressed as the number of detected viral copies in 5 μL of extracted RNA. In RT-qPCR for specific virus detection, the tested samples were interpreted as negative if no cycle threshold (Ct) was detected in samples (Ct > 45). The samples were interpreted as very weak positive when between 1 and 100 copies were detected in 5 μL of virus RNA. The samples were interpreted as weak positive when between 101 and 1000 copies were detected in 5 μL of virus RNA. The samples were interpreted as positive when between 1001 and 100,000 copies were detected in 5 μL of virus RNA. The samples were interpreted as strong positive when more than 100,001 copies were detected in 5 μL of virus RNA.

### 2.6. Presence and Quantification of Mites V. destructor

All 39 collected samples of winter debris were examined for the presence and quantification of the *V. destructor* mite. According to medical history, each honeybee colony underwent treatment with authorized Veterinary Medicinal Products (VMPs) against varroosis in the previous active beekeeping season. By weighing each sample, its weight in grams is determined. The number of mites from each honeybee colony was recorded in different sample quantities, which is why each result was converted to the mean of *V. destructor* mites in one gram of debris.

### 2.7. Clinical Examination of Honeybee Colonies in the Spring and Determination of the Natural Fall of V. destructor Mites

In spring 2022 (5–25 April), all honeybee colonies were clinically examined. All inspections were performed by same person. The aim was to determine the strength of the honeybee colonies and the possible presence of clinical signs characteristic of a particular disease. The natural fall of *V. destructor* was also determined in the active beekeeping season before treatment with acaricides. Before the fallen *V. destructor* mites were counted, each bottom board was cleaned. The number of fallen mites was recorded on the first, fifth, and seventh day.

## 3. Results

Using the conventional PCR method, *V. ceranae* (*N. ceranae*) was determined in 87.2% (34/39) of the examined winter hive samples. The presence of *V. apis* (*N. apis*) was not detected in any of the samples. Four samples were unsuitable for interpretation.

**Figure 1:**
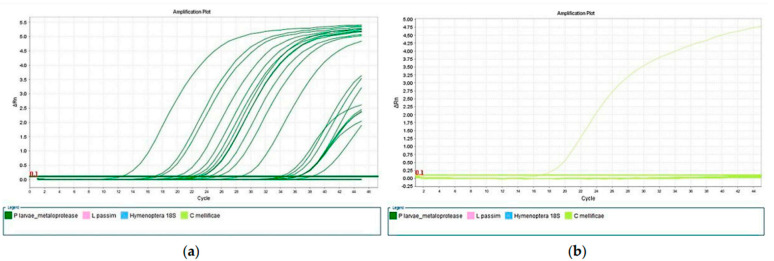
Examination results for the presence and quantification of *P. larvae* (**a**) and *C. mellificae* (**b**) using real-time PCR methods.

*P. larvae* were found in 17 of the 39 samples examined (43.6%). Only in A6 was no infection with *P. larvae* detected in any sample, while it was confirmed in all other apiaries (Figure 1a). In the following spring, the clinical signs characteristic of American foulbrood appeared only in A2.

On conducting real-time PCR methods for determining *C. mellificae*, *L. passim* (Figure 1b), *M. plutonius*, and *A. tumida*, no positive samples were found.

**Figure 2:**
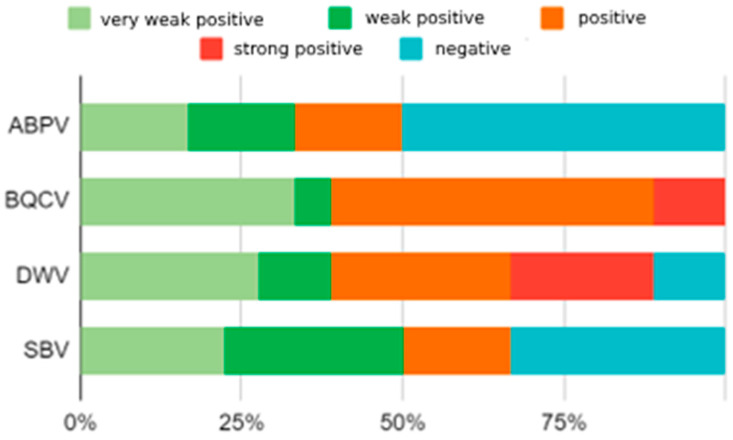
The results of ABPV, BQCV, DWV and SBV detection by specific RT-qPCRs in the tested samples of hive debris. Legend: Negative sample: no viral RNA was detected in tested samples: less than 1 copy of virus RNA in 5 μL of tested RNA; Very weak positive sample: between 1 and 100 copies of virus RNA were detected in 5 μL of tested RNA; Weak positive sample: between 101 and 1000 copies of virus RNA were detected in 5 μL of tested RNA; Positive sample: between 1001 and 100,000 copies of virus RNA were detected in 5 μL of tested RNA; Strong positive sample: more than 100,001 copies of virus RNA were detected in 5 μL of tested RNA.

**Figure 3:**
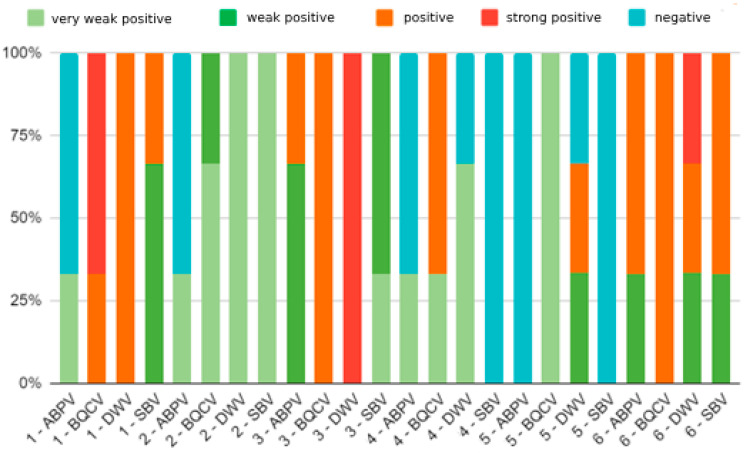
The summarised results of detected percentages of ABPV, BQCV, DWV and SBV, by specific RT-qPCRs in the tested samples of hive debris. Legend: Negative sample: no viral RNA was detected in tested samples: less than 1 copy of virus RNA in 5 μL of tested RNA; Very weak positive sample: between 1 and 100 copies of virus RNA were detected in 5 μL of tested RNA; Weak positive sample: between 101 and 1000 copies of virus RNA were detected in 5 μL of tested RNA; Positive sample: between 1001 and 100,000 copies of virus RNA were detected in 5 μL of tested RNA; Strong positive sample: more than 100,001 copies of virus RNA were detected in 5 μL of tested RNA.

Out of 18 samples searched, 9 samples (50%) were positive for the presence of ABPV. Furthermore, the presence of BQCV was found in all analyzed samples (100%). The samples were positive for DWV in 83.3% of cases (15/18) and for the SBV in 66.6% (12/18). The results for viral pathogens were divided, according to the established loads, into five groups: negative, very weakly positive, weakly positive, positive, and very positive (Figure 2). In 45% of honeybee colonies, low levels of infection were found, and the remaining 35% of positive samples were found to have a high level of infection. We found that in most honeybee colonies several viral pathogens were present at the same time. That is, most colonies tested positive for all four examined viruses, except A4, which is free of the SBV virus, and A5, which is free of the SBV and ABPV (Figure 3) Observed honeybee colonies that did not survive the winter of 2022/2023 showed a high level of simultaneous infection with BQCV and DWV.

**Figure 4:**
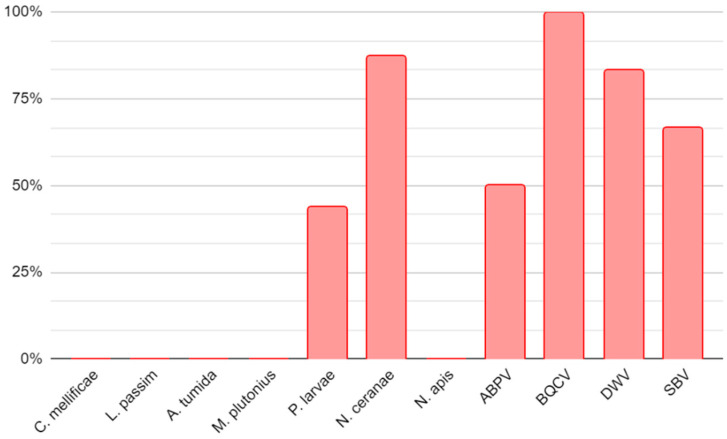
An overview of the identified pathogens in debris samples taken from bottom boards of hives.

An overview of the identified pathogens in winter hive debris samples is shown in Figure 4.

**Figure 5:**
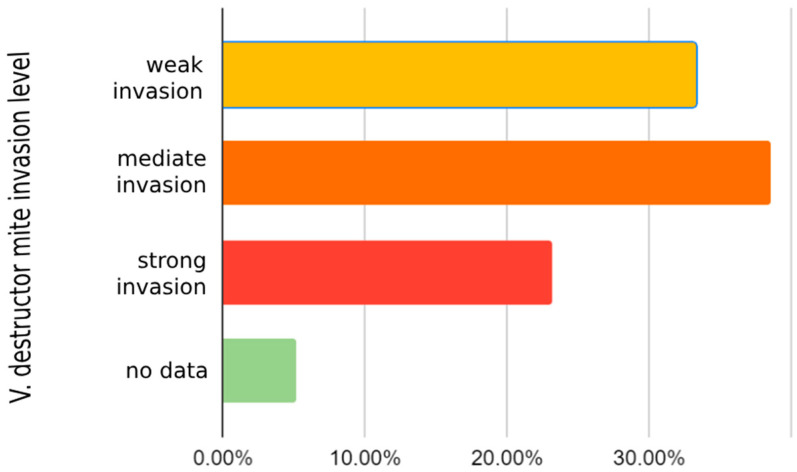
The proportion of parasitic mites *V. destructor* in one gram of hive debris sample, taken in the winter period at all observed apiaries.

**Figure 6:**
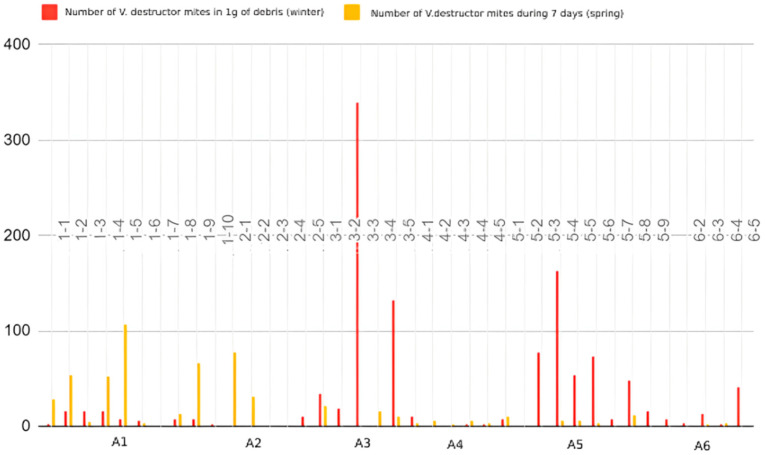
The number of counted parasitic mites *V. destructor* from each observed hive and from hive bottom board debris samples, collected in winter and spring.

By determining the number of fallen *V. destructor* mites during wintering, hives were divided into three groups: low, medium and heavily infested. The group of low-infested honeybee colonies belongs to 33.3% (13/39) of the examined hive debris samples that are most present in A2 and A4, the group of medium-infested belongs to 38.5% (15/39) of hive debris samples, which are mostly located in A1 and A6, and in 23.1% (9/39) hives a strong infestation was found. In winter hive debris samples, the largest infestation was recorded in A3 with 339 and A5 with 162.2 counted mites in one gram of debris sample. The results obtained from the two samples were unsuitable for interpretation (Figure 5). In the spring, the natural fall counting of the *V. destructor* mite during seven days determined the largest infestation in A1 and A2 (Figure 6).

In A1, an average of six to seven pathogens were found within a single honeybee colony (Table 1). All the observed honeybee colonies demonstrated positivity for *P. larvae*, but no clinical signs of American foulbrood were detectable in the spring. It is noteworthy that honeybee colonies that did not survive the winter had a higher number of *P. larvae* spores than those that survived. Two honeybee colonies (A1–7 and A1–10) were found dead (marked in red in Table 1), and analysis showed that they were infected with *P. larvae* and *V. ceranae* (*N. ceranae*), along with confirmed DWV infection and a high BQCV level. Additionally, the A1–10 hive had high levels of SBV. It is interesting to note that both honeybee colonies were part of the group with low mite infestation with *V. destructor*. While the winter debris showed weak and medium levels of infestation, A1 had the highest infestation of honeybee colonies in the spring. All colonies tested positive for *V. ceranae* (*N. ceranae*), but *V. apis* (*N. apis*) was not detected in any sample. During spring, there were no apparent clinical signs of Vairimorphosis (Nosemosis). Surviving honeybee colonies had an average of six to seven brood combs and an average of 22 frame spaces occupied by adult bees. The A1–2 hive had a weak colony with only eight filled frame spaces with adult bees, and there were visible clinical signs of disease, like spotty pattern brood. The A1–3 hive had an extremely weak colony with only two frames containing drone brood.

In A2 an average of five to six pathogens were found in one honeybee colony (Table 2). Among them, a lower load of *P. larvae* was observed, and in the spring, in hives A2–3, A2–4, and A2–5 (marked in red in Table 2), clinical signs of American foulbrood were detected, and they were eradicated following the applicable prescribed measures. Only one hive debris sample did not contain *P. larvae*. All colonies tested positive for *V. ceranae* (*N. ceranae*), while *V. apis* (*N. apis*) were not found in any sample. During the spring season, there were no visible signs of Vairimorphosis (Nosemosis). However, upon examining the number of *V. destructor* present in the hive winter debris, it was discovered that most hives belonged to the low-infestation group, except for the A2–5 hive, which had a medium level of infestation. Despite this, in the spring, it was found that the surviving honeybee colonies were heavily infested. On average, the two surviving honeybee colonies had eight brood frames and adult bees occupied an average of 22 frame spaces.

In A3, an average of five to six disease-causative agents were found in a single honeybee colony (Table 3). In honeybee colonies that tested positive for *P. larvae*, low levels of infestation were reported. Unfortunately, two colonies did not survive the winter (marked in red in Table 3), one of which tested negative for *P. larvae* but positive for *V. ceranae* (*N. ceranae*) with an extremely high degree of virus infection, including DWV and BQCV. The other honeybee colony had a lower load of *P. larvae* but an extremely high infestation of *V. destructor* mites and a high titer of DWV and BQCV. A hive with the largest infestation of *V. destructor* mites was recorded in the A3–3 debris sample that did not survive the winter, with 339 counted mites. In the surviving colonies, the median level of varroa mites infestation prevailed in the spring. The surviving honeybee colonies had an average of eight brood frames and 18 frame spaces occupied by adult bees. Clinical signs of ABPV and DWV were observed in the spring, but there were no visible characteristic signs of American foulbrood. 

In A4, an average of four to five pathogens were found in one honeybee colony, in samples that were also tested for viruses (Table 4). During clinical examination in the spring, it was found that all honeybee colonies had survived the winter. Three honeybee colonies tested negative for *P. larvae*, while low levels of *P. larvae* were detected in the remaining two colonies. However, no clinical disease development was observed in the spring. Four colonies tested positive for *V. ceranae* (*N. ceranae*), while one sample was unsuitable for interpretation. No visible clinical signs of Vairimorphosis (Nosemosis) were observed in the spring. A strong infestation of *V. destructor* was detected in three hives by determining the number of mites in winter hive debris. However, the infestation was weak in the spring. The most prevalent viral agent found was BQCV, which caused visible clinical signs in hive A4–1 during the spring. On average, adult bees occupied 20 frame spaces and nine brood frames each.

In A5, on average, three to four pathogens were found in a single honeybee colony (Table 5). All colonies survived the winter, and only one tested positive for *P. larvae*, which did not result in clinical disease. Most honeybee colonies were infected with *V. ceranae* (*N. ceranae*), while one was negative, and two debris samples were unsuitable for interpretation. A high infestation of *V. destructor* was detected in four hives from winter debris, but the same hives were only low to medium infested in the spring. DWV had the highest level among the viral causes, and sporadic clinical signs of this virus were present in the spring. No honeybee colonies tested positive for ABPV and SBV. In spring, adult bees occupied approximately 20 hive frame spaces and 9 brood frames on average, except for hive A5–7, which had a weaker honeybee colony, with only 9 frame spaces of adult bees and 5 brood frames.

In A6, an average of five to six pathogens were recorded within one honeybee colony (Table 6). All honeybee colonies were negative for the presence of *P. larvae*, and positive for *V. ceranae* (*N. ceranae*), except for one whose analyzed result was unsuitable for interpretation. In the spring, no signs of Vairimorphosis (Nosemosis) were visible. By determining the number of *V. destructor* from winter debris samples, a low infestation in four hives and a medium level of infestation in the hive marked A6–1 was determined, and in the spring the lowest number of mites of all monitored apiaries was detected. In samples from three hives, high levels of all four viruses were found. A clinical examination of A6 in the spring found that one colony had died (marked in red in Table 6). The strength of honeybee colonies was weak, with an average of seven brood frames and ten frame spaces occupied by adult bees. Among the clinical signs were visible signs of the DWV.

## 4. Discussion

This study represents not only the first investigation on the occurrence of various pathogens in samples of winter hive debris in Croatia, but it is also one of the few that analyses the congruence between the health status of honeybee colonies and results obtained in laboratory research. Although there is a lack of data on the reliability/sensitivity/diagnostic value of winter hive debris as a sample for the determination of the health status of the honeybee colony, there are also some known advantages, i.e., the sampling is non-invasive, it is easy to perform and is accessible regardless of the season. 

In doing so, the presence and/or absence of individual pathogens or pests using the PCR/qPCR method was determined, and the results were compared with the health status and strength of honeybee colonies in the spring. Although sampling of honey, suspected/altered brood, and adult bees are often used in everyday practice, such sampling also carries some drawbacks. Namely, sampling of material from the hive intended for laboratory research takes a lot of time since it is necessary to open each hive, and official sampling is carried out by an authorized veterinarian. Furthermore, any opening of the hive causes an increase in stress for the honeybee colony. In addition, the possibility of opening the hive also depends on weather conditions. On the other hand, debris from the hive bottom boards can be collected very easily in any weather conditions, which makes it possible to analyze the state of health of the honeybee colony in the winter months. A beekeeper can also take samples. A sample examination can determine the presence of a causative disease agent at one or more apiaries during the winter months and can help in timely taking control and/or preventive measures to avoid visible signs of a particular disease, and thus of the outbreak of the disease and its spread during the active beekeeping season. 

In practice, the coprological method is often used to diagnose Vairimorphosis (Nosemosis), which requires sacrificing adult bees. However, we were able to successfully determine the presence of the causative agent of this disease using non-invasive sampling of winter hive debris. Copley et al. (2012) also confirmed that evaluation of debris samples is just as reliable as analyzing samples of adult bees [35]. We were able to determine the presence only of *N. ceranae* as the dominant species, which is consistent with previous findings [36], which concluded that *N. ceranae* is the only species of the genus *Nosema* spp. present in the territory of Croatia. Since we did not observe visible signs of diarrhea in early spring in all laboratory-confirmed positive honeybee colonies, we can confirm that infection with *N. ceranae* is an asymptomatic disease, or in rare cases shows non-specific symptoms.

In this study, we confirmed that the investigation of winter debris samples using the quantitative PCR method is useful for the early detection of *P. larvae* infection in honeybee colonies. Forsgren and Laugen (2014) found significantly more spores in the winter debris than in samples of adult bees using molecular and culturing methods [27], probably because in winter debris *P. larvae* spores accumulate for months, unlike in a sample of adult live bees whose lifespan lasts several weeks. Using the PCR method for analyzing samples of winter debris, the highest loads of *P. larvae* spores were determined in hives where subsequent inspections of honeybee colonies show the highest proportion of clinically visible symptoms characteristic of American foulbrood (in 40 out of 58 clinically inspected honeybees colonies). The same authors concluded that PCR analysis of accumulated hive debris is the best method for the detection of subclinical loads of *P. larvae*. However, their results also showed that the culture-based method is more accurate for detecting the causative agent in clinically diseased honeybee colonies and predicting colony health status from diagnostic results generated from adult bee samples. According to the results of this study, we also confirmed that the presence of *P. larvae* in winter debris does not mean the sure occurrence of a clinically visible symptoms characteristic to American foulbrood, since low levels of spores can be frequently determined in different hive materials. Of the 17 positive samples, only three honeybee colonies developed characteristic clinical signs of the disease, found during spring inspections. It is known that the development of clinical signs of the disease depends on several factors, such as the strength of the honeybee colony, its hygienic behavior, the ERIC type of *P. larvae*, and implemented beekeeper practices [37,38]. For those reasons, further research is needed to examine more precisely the relations between the method of debris sampling, the number of spores found in winter debris and the risk of developing of clinical form of the disease for a more accurate early diagnosis of American foulbrood. In everyday veterinary inspection practice, only honeybee colonies with visible disease symptoms should be eliminated in the frame of eradication and biosecurity measures at the apiary.

Biova et al. (2021) proved that from the hive debris, the presence of the bacterium *M. plutonius* can be determined [26]. Although samples of honey and adult bees yielded more reliable results, they concluded that samples of hive debris are more effective for non-invasive monitoring of European foulbrood. However, according to the results obtained from this study, not a single positive sample of *M. plutonius* was found in the examined apiaries.

Although trypanosomatids are very common within honeybee colonies, no positive debris samples have been confirmed during this study. For this reason, it is necessary to sample several apiaries from different locations and to compare debris samples with samples of adult bees to obtain more objective results and assess the suitability of debris for testing for such pathogens. It may be useful to try alternative methods of DNA extraction, such as a single-tube method that reduces DNA loss, and to experiment with different primers for *C. mellificae* and *L. passim*.

By analyzing winter debris, as expected, not a single positive sample of *A. tumida* has been confirmed since Croatia is still free from small hive beetle invasion. However, we have proven that analyzing for such samples using the qPCR method is extremely useful for a rapid diagnosis of its presence in new geographical areas or where it is present at low levels, which is of great importance for timely routine monitoring of hives in high-risk areas.

Many studies have been carried out on the topic of virus diagnostics in honeybees, in one of them in 2018, an analysis of debris was performed for the presence of eight viruses, including ABPV, BQCV, SBV and DWV. According to the results of these studies, in most cases, viral pathogens were confirmed more precisely than in samples of adult bees or larvae, which is why the authors concluded that samples of debris could be a better indicator of hygiene within the honeybee colony [39]. In the current study, we have determined that winter debris is a suitable, non-invasive material for determining the presence of viral pathogens. By real-time RT-PCR test, we found that most honeybee colonies are simultaneously infected with several viruses. We found a high infection of honeybee colonies with black BQCV (100% of samples) and DWV (83.3%), while infection with SBV is slightly lower (66.6%), and with ABPV the lowest (50%). It is interesting to note that winter losses of honeybee colonies in this study were strongly associated with high levels of DWV and BQCV in the honeybee colony, except for three colonies in A2 that were destroyed by the eradication of the clinical form of American foulbrood.

The number of *V. destructor* detected by analyzing winter debris also provided a valuable method of monitoring the level of honeybee colony infestation by this parasite during the winter months. In several honeybee colonies, we detected a very high number of *V. destructor* mites in 1 g of winter debris, while the natural fall in spring was low. This was probably the result of effective winter treatment against Varroosis, which is why fewer mites were present in the honeybee colony in spring. We also found some honeybee colonies with low numbers of varroa mites in the winter debris that had a high natural fall in the spring. We suspect that in these honeybee colonies, the winter treatment was not carried out or was not effective, so the number of varroa mites was high in the spring. 

A limitation of this study is that we do not know the history of anti-varroa treatment in these honeybee colonies. For this reason, we do not know whether the increased number of *V. destructor* mites in the hive debris the result of a high infestation or an ineffective treatment is. However, counting those parasitic mites in the winter hive debris is a useful method to determine infestation levels, but it is necessary to consider data on previous treatments. By linking the number of *V. destructor* mites counted in spring and honeybee colonies’ strength, most highly invaded honeybee colonies were weaker and seemed slower in spring development.

## 5. Conclusions

Winter hive debris analysis is a non-invasive, simple, and cost-effective method for detecting the presence of various pathogens. The early detection of subclinical levels of *P. larvae* in winter debris is a valuable technique in preventing the spread of the disease in spring. In our study, *V. ceranae* (*N. ceranae*) was the only confirmed species of the genus *Vairimorpha* (*Nosema*) and the most frequently found pathogen. Honeybee colonies were simultaneously infected with multiple pathogens and pests. On average, honeybee colonies were infected with four to six pathogens, with BQCV and DWV being the most commonly identified viruses. The level of infestation of the *V. destructor* mite can also be determined by analyzing winter hive debris samples.

## Figures and Tables

**Table 1:** Summarized results of laboratory examinations of hive debris samples originating from A1.

Hive	*P. larvae* (Ct)	*Vairimorpha* (*Nosema*) spp.	Number of *V. destructor* Mites in 1 g Sample (Winter)	Number of *V. destructor* in 7 Days (Spring)	ABPV	BQCV	DWV	SBV	Number of Frames Bees/Brood
A1–1	25, 45	*N. ceranae*	2.15	29	very weak positive	positive	positive	weak positive	22/7
A1–2	17, 36	*N. ceranae*	16.25	54	-	-	-	-	8/0
A1–3	24, 24	*N. ceranae*	15.6	5	-	-	-	-	2/2
A1–4	21, 26	*N. ceranae*	16.6	52	-	-	-	-	23/7
A1–5	22, 61	*N. ceranae*	8	107	-	-	-	-	22/6
A1–6	23, 03	*N. ceranae*	6.8	3	-	-	-	-	22/7
A1–7	16, 82	*N. ceranae*	0.2	-	negative	strong positive	positive	weak positive	0/0
A1–8	22, 97	*N. ceranae*	8.2	13	-	-	-	-	22/6
A1–9	22, 03	*N. ceranae*	7.5	66	-	-	-	-	22/7
A1–10	12, 37	*N. ceranae*	2.3	-	negative	strong positive	positive	positive	0/0

-, not tested.

**Table 2:** Summarized results of laboratory examinations of hive debris samples originating from A2.

Hive	*P. larvae* (Ct)	*Vairimorpha* (*Nosema*) spp.	Number of *V. destructor* Mites in 1 g Sample (Winter)	Number of *V. destructor* in 7 Days (Spring)	ABPV	BQCV	DWV	SBV	Number of Frames Bees/Brood
A2–1	33, 98	*N. ceranae*	1.3	78	negative	very weak positive	very weak positive	very weak positive	10/22
A2–2	negative	*N. ceranae*	0.5	32	negative	very weak positive	very weak positive	very weak positive	6/22
A2–3	38, 25	*N. ceranae*	0.4	-	very weak positive	weak positive	very weak positive	very weak positive	0/0
A2–4	36, 3	*N. ceranae*	0.1	-	-	-	-	-	0/0
A2–5	36, 29	*N. ceranae*	10.5	-	-	-	-	-	0/0

-, not tested.

**Table 3:** Summarized results of laboratory examinations of hive debris samples originating from A3.

Hive	*P. larvae* (Ct)	*Vairimorpha* (*Nosema*) spp.	No. *V. destructor* Mites in 1 g Sample (Winter)	No. *V. destructor* in 7 Days (Spring)	ABPV	BQCV	DWV	SBV	Number of Frames Bees/Brood
A3–1	28, 53	*N. ceranae*	34	21	positive	positive	strong positive	weak positive	9/20
A3–2	negative	*N. ceranae*	18.6	-	weak positive	positive	strong positive	weak positive	0/0
A3–3	35, 06	*N. ceranae*	339	-	weak positive	positive	strong positive	very weak positive	0/0
A3–4	34, 46	*N. ceranae*	-	16	-	-	-	-	10/18
A3–5	negative	*N. ceranae*	132.5	11	-	-	-	-	5/16

-, not tested.

**Table 4:** Summarized results of laboratory examinations of hive debris samples originating from A4.

Hive	*P. larvae* (Ct)	*Vairimorpha* (*Nosema*) spp.	No. *V. destructor* Mites in 1 g Sample (Winter)	No. *V. destructor* in 7 Days (Spring)	ABPV	BQCV	DWV	SBV	Number of Frames Bees/Brood
A4–1	negative	*N. ceranae*	7	4	very weak positive	positive	negative	negative	22/10
A4–2	36, 25	*N. ceranae*	1	6	negative	positive	very weak positive	negative	22/10
A4–3	36, 28	*N. ceranae*	77.2	2	negative	very weak positive	very weak positive	negative	18/10
A4–4	negative	*N. ceranae*	162.6	6	-	-	-	-	16/8
A4–5	negative	unsuitable for interpretation	54	4	-	-	-	-	20/9

-, not tested.

**Table 5:** Summarized results of laboratory examinations of hive debris samples originating from A5.

Hive	*P. larvae* (Ct)	*Vairimorpha* (*Nosema*) spp.	No. *V. destructor* Mites in 1 g Sample (Winter)	No. *V. destructor* in 7 Days (Spring)	ABPV	BQCV	DWV	SBV	Number of Frames Bees/Brood
A5–1	36, 23	unsuitable for interpretation	74	11	negative	very weak positive	positive	negative	18/6
A5–2	negative	negative	7.25	1	negative	very weak positive	weak positive	negative	22/10
A5–3	negative	*N. ceranae*	48.5	1	-	-	-	-	20/10
A5–4	negative	*N. ceranae*	16.3	6	-	-	-	-	22/10
A5–5	negative	*N. ceranae*	7	6	-	-	-	-	20/9
A5–6	negative	*N. ceranae*	3.6	3	-	-	-	-	20/10
A5–7	negative	*N. ceranae*	13	1	negative	very weak positive	negative	negative	9/5
A5–8	negative	*N. ceranae*	2.5	12	-	-	-	-	20/12
A5–9	negative	unsuitable for interpretation	41	0	-	-	-	-	22/10

-, not tested.

**Table 6:** Summarized results of laboratory examinations of hive debris samples originating from A6.

Hive	*P. larvae* (Ct)	*Vairimorpha* (*Nosema*) spp.	No. *V. destructor* Mites in 1 g Sample (Winter)	No. *V. destructor* in 7 Days (Spring)	ABPV	BQCV	DWV	SBV	Number of Frames Bees/Brood
A6–1	negative	*N. ceranae*	10.8	0	positive	positive	positive	positive	10/8
A6–2	negative	unsuitable for interpretation	no data	0	positive	positive	weak positive	positive	10/6
A6–3	negative	*N. ceranae*	0.73	-	weak positive	positive	strong positive	weak positive	0/0
A6–4	negative	*N. ceranae*	2.3	3	-	-	-	-	10/7
A6–5	negative	*N. ceranae*	2.6	0	-	-	-	-	10/7

-, not tested.

## Data Availability

Data were presented in the manuscript.

## Supplementary Materials

The following supporting information can be downloaded at: https://www.mdpi.com/article/10.3390/insects15050350/s1, Table S1: Presentation of specific oligonucleotide primers for determining *Nosema* spp. [30]. Table S2: Presentation of specific oligonucleotide primers for the determination of *C. mellificae* and *L. passim* with the Hymenoptera genome as internal control [25]. Table S3: Presentation of TaqMan probes for determining *C. mellificae* and *L. passim* with the Hymenoptera gene as an internal control [25]. Table S4: Presentation of specific oligonucleotide primers for determining *M. plutonius* and *P. larvae* [31]. Table S5: Presentation of TaqMan probes used to determine *M. plutonius* and *P. larvae* [31]. Table S6: Presentation of specific oligonucleotide primers for determining *A. tumida* [32]. Table S7: Presentation of TaqMan probes used to determine *A. tumida* [32]. Table S8: Sequences of primers used for the RT-qPCR test [34]. Table S9: TaqMan probes used for RT-qPCR test [34]. Table S10: Standards used to quantify examined viruses by RT-qPCR test [34].
