# Supplementary material for: Winter Hive Debris Analysis Is Significant for Assessing the Health Status of Honeybee Colonies (Apis mellifera)
Source: Insects. 2024 May 13;15(5):350. doi: 10.3390/insects15050350 (PMC11121827; doi:10.3390/insects15050350)
Supplement: Supplementary file 1 [file insects-15-00350-s001.zip › insects-2993670-supplementary.pdf]

Table S1 Presentation of specific oligonucleotide primers for determining *Nosema* spp. [30].

Species	Nucleotide sequence of upstream primers 5'-3'	Nucleotide sequence of downstream primer 5'-3'	Size
Nosema ceranae	CGT TAA AGT GTA GAT AAG ATG TT	GAC TTA GTA GCC GTC TCT C	143 bp
Nosema apis	GCA TGT CTT TGA CGT ACT ATG	GAC TTA GTA GCC GTC TCT C	224 bp

Table S2 Presentation of specific oligonucleotide primers for the determination of *C. mellificae* and *L. passim* with the Hymenoptera genome as internal control [25].

Species	Nucleotide sequence of upstream primers 5'-3'	Nucleotide sequence of downstream primers 5'-3'
Crithidia mellificae	TAA ATT CAC TAC CTC AAA TTC AAT AAC ATA ATC AT	ATT TAT TGT TGT AAT CGG TTT TAT TGG ATA TGT
Lotmaria passim	CGA GCT CAT AAA ATA ATG TAA GCA AAA TAA G	TTT TAG CAA TAT TTT AGC AAC AGT ACC AG
Hymenoptera	TAA CTG GCA TTA TGT GGT ACG TC	CCT CGA CAC TCA GTG AAG AGC

Table S3 Presentation of TaqMan probes for determining *C. mellificae* and *L. passim* with the Hymenoptera gene as an internal control [25].

Species	TaqMan probes
Crithidia mellificae	FAM - ACC TAT TAC AGG CAC A - MGB
Lotmaria passim	HEX - TTG GTG TTT GGC TAT GT - MGB
Hymenoptera	Cy5 - AGC TCC TYG CGG GCG GTC CAA - BHQ1

Table S4 Presentation of specific oligonucleotide primers for determining *M. plutonius* and *P. larvae* [31].

Species	Nucleotide sequence of upstream primers 5'-3'	Nucleotide sequence of downstream primers 5'-3'
Melissococcus plutonius	GAC CTG TTT AGC TAT TAT CAC TA	CAC CTA CAA TGA ATG ATT CAT TC
Paenibacillus larvae	TAC GCT TTT CGA TTC TCT G	GTC TGT ACT GAA CCA AGT C

Table S5 Presentation of TaqMan probes used to determine *M. plutonius* and *P. larvae* [31].

Species	TaqMan probes
Melissococcus plutonius	Yakima Yellow – TCC GCC TAA GCT ACC ACC TAA GAA C - BHQ1
Paenibacillus larvae	FAM – ATC TGC TTC CAC TTG TTC ACT CAC CA - BHQ1

Table S6 Presentation of specific oligonucleotide primers for determining *A. tumida* [32].

Species	Nucleotide sequence of upstream primers 5'-3'	Nucleotide sequence of downstream primers 5'-3'
Aethina tumida	TCT AAA TAC TAC TTT CTT CGA CCC ATC(A/G)	TCC TGG TAG AAT TAA AAT ATA AAC TTC TGG

Table S7 Presentation of TaqMan probes used to determine *A. tumida* [32].

Species	TaqMan probe
Aethina tumida	ATC CAA TCC TAT ACC AAC ACT TAT TTT GAT TCT TCG GAC

Table S8 Sequences of primers used for the RT-qPCR test [34].

Species	Nucleotide sequence of upstream beginners 5'-3'	Nucleotide sequence of upstream primer 5'-3'
ABPV	CAT ATT GGC GAG CCA CTA TG	CTA CCA GGT TCA AAG AA ATT TC
BQCV	GGT GCG GGA GAT GAT ATA TGG A	GCC GTC TGA GAT GCA TGA ATA C
DVW	GCG GCT AAG ATT GTA AAT GTC	GTG ACT AGC ATA ACC ATG ATT A
SBV	AGC CAG TGA TAG ATG CTC	AAA TAC TCC CGC CAA ATC AC

Table S9 TaqMan probes used for RT-qPCR test [34].

Species	TaqMan probe (5'-3')
ABPV	(6-Fam) ATA GTT AAA ACA GCT TTT CAC ACT GG (Tamra)
BQCV	(6-Fam) TTT CCA TCT TTA TCG GTA CGC CGC C (Tamra)
DWV	(6-Fam) CCT TGA CCA GTA GAC AGC ATC (Tamra)
SBV	(6-Fam) TGG CTC ATC TGG GAT CAC AAT TTC C (Tamra)

Table S10 Standards used to quantify examined viruses by RT-qPCR test [34].

Species	Standard	Nucleotide position	Sequence position
ABPV	pB2	8115 - 8512	397
BQCV	pNC1-4	7850 - 8550	700
DWV	pC1	4240 - 4659	419
SBV	SBV	5030 - 5368	338
